# Association of the Plasma and Tissue Riboflavin Levels with C20orf54 Expression in Cervical Lesions and Its Relationship to HPV16 Infection

**DOI:** 10.1371/journal.pone.0079937

**Published:** 2013-11-18

**Authors:** Aixingzi Aili, Ayshamgul Hasim, Alimujiang Kelimu, Xia Guo, Batur Mamtimin, Abuliz Abudula, Halmurat Upur

**Affiliations:** 1 Department of Gynecology, the First Affiliated Hospital of Xinjiang Medical University, Urumqi, Xinjiang, China; 2 Department of Pathology of the Medical University of Xinjiang, Urumqi, Xinjiang, China; 3 Department of Neurosurgery, First Affiliated Hospital of Xinjiang Medical University, Urumqi, Xinjiang, China; 4 Xinjiang Medical University, Urumqi, Xinjiang, China; 5 Pharmaceutical College of Xinjiang Medical University, Urumqi, Xinjiang, China; Baylor College of Medicine, United States of America

## Abstract

Riboflavin deficiency can cause a variety of metabolic problems that lead to skin and mucosal disorders. Limited evidence suggests that high intake of riboflavin may reduce overall risks of cancer. However, association of this deficiency with cervical cancer and precancerous lesions are still not definitively known. In this study, we characterized the relationship between plasma and tissue riboflavin levels and C20orf54 protein expression in patients with cervical intraepithelial neoplasia (CIN) and cervical squamous cell carcinoma (CSCC) as well as the relationship of these levels with human papillomavirus virus 16, 18 (HPV16/18) infections. High-performance liquid chromatography (HPLC) was used to measure blood riboflavin levels in patients with CIN and CSCC, and an enzyme-linked immunosorbent assay (ELISA) was used to determine tissue riboflavin levels in patients with CSCC and matched normal mucous epithelia. The expression of C20orf54 in fresh CSCC and matched tissues were detected by qRT-PCR and western blot, respectively. And it was further confirmed by immunohistochemistry (IHC) with formalin-fixed, paraffin-embedded CIN and CSCC. An HPV genotyping chip was used to analyze HPV infection and typing. The results showed that patients with CIN and CSCC had decreased plasma riboflavin levels as compared with normal controls. There was also significantly decreased riboflavin in tissues from CSCC patients, when compared with normal cervical epithelia. C20orf54 expression were significantly up-regulated in CSCC compared to matched control on both mRNA and protein level. Tissue riboflavin levels were significantly lower in HPV16/18 positive tissue compared with HPV16/18-negative tissue, and an inverse association was found between tissue riboflavin levels and C20orf54 mRNA and protein expression in CSCC. Additionally, C20orf54 was significantly correlated with tumor stages. In conclusion, C20orf54 tend to play a protective role in Uyghur cervical carcinogenesis of which modulating riboflavin absorption, and it is also related with HPV infection.

## Introduction

Worldwide, cervical cancer has the second highest incidence of cancer in women. Human papillomavirus (HPV) infection plays a central role in the pathogenesis of cervical cancer and its precancerous lesions (CIN), with HPV infection considered a necessary, but not always sufficient, cause [1]–[2]. Development of human cervical cancer without involvement of a specific HPV is exceptional, but it is widely accepted that in addition to HPV infection, other cofactors may have important roles in the development of cervical lesions [3]–[4]. HPV infection is usually transient, and only a small proportion of women who test positive for high risk HPV infection actually develop cervical cancer. Therefore, HPV infection may be necessary but not sufficient to cause cervical cancer. In addition, nutritional factors may also affect the persistence of HPV infection and thereby influence the progression of early precancerous lesions to invasive cancer [5]–[6].

Riboflavin (vitamin B2) is an essential vitamin that is required for normal cellular functions, including growth and development in all aerobic forms of life. It is a water soluble vitamin that occurs in two major forms, flavin mononucleotide (FMN) and flavin adenine dinucleotide (FAD). It also participates in various metabolic redox reactions, and is involved in one carbon metabolism, which is a network of interrelated biochemical pathways that generate one carbon groups needed for physiologic processes [7]–[9]. Disruption of one carbon metabolism can interfere with DNA replication, DNA repair, and regulation of gene expression through methylation, each of which could promote carcinogenesis [10]. It has been reported that deficiency of riboflavin plays a prominent role in progression of various cancers as well as increased vulnerability of cells to cancer [11]. In addition, it has been implicated in the enhancement of antitumor activity of many anticancer drugs, as well as in activation of the immune system to kill tumor cells [12]–[14].

C20orf54 is a human riboflavin transporter that has an important role in the intestinal absorption of riboflavin [15]–[16], and riboflavin deficiency has been associated with an increased risk of esophageal squamous cell carcinoma (ESCC) and gastric cardia adenocarcinoma (GCA). Riboflavin supplementation has been reported to reduce the risk of ESCC and GCA [17]–[18], so C20orf54 may have an important role in modulating riboflavin absorption.

Cervical cancer in Uyghur occurs with high morbidity and mortality in women in the Xinjiang region, which is considered a high incidence disease region in China [19]–[20]. Some epidemiologic studies have reported a relationship between cervical cancer and diets low in riboflavin [21]–[22], and animal studies have shown that riboflavin deficiency can lead to disruption of the esophagus epithelium, in a similar manner to precancerous lesions in humans [23]. Poor riboflavin status has also been reported as a risk factor for cervical dysplasia, a precursor condition for invasive cervical cancer [24]. However, in the Uyghur population, extensive consumption of milk and dairy products, meats, fatty fish, certain fruit, and vegetables are good sources of riboflavin. Thus, whether other factors exist that may influence dietary riboflavin absorption or that may change the status of riboflavin needs to be determined.

Based upon these findings, we hypothesize that human C20orf54 plays an important role in cervical carcinogenesis involving modulation of riboflavin absorption. This study therefore investigated blood and tissue riboflavin levels, as well as the status of the riboflavin transporter (C20orf54) gene, in cervical cancer patients and cervical intraepithelial neoplasia patients living in Uyghur.

## Materials and Methods

### Clinical Characteristics and Samples

Formalin-fixed, paraffin-embedded (FFPE) and fresh frozen cervical tissue specimens were collected from Uyghur women with CSCC and CIN, or matched normal mucous epithelia collected 5 cm away from the tumor. Peripheral blood samples were also collected from patients described above. All cervical cancer patients referred between July 2010 and May 2012 because of cervical cancer were asked to participate in our study during their initial visit to the Department of Gynecology of the First Affiliated Hospital in the Medical University of Xinjiang. Written informed consent was obtained from all patients and controls participating in this study, and the study were approved by the ethics committee of first affiliated hospital of Xinxiang medical university. Gynecological examination was performed for all cervical cancer patients for staging in accordance with the International Federation of Gynecology and Obstetrics (FIGO) criteria.

A total of 146 FFPE tissue specimens consisting of 50 cases CSCC and 50 cases matched normal mucous epithelia, and 46 cases CIN patients were obtained from the archive of the Pathology Department after examination of archival slides by experienced pathologists, which used to measure C20orf54 protein expression. Of patients with CSCC enrolled in this study, were 22 FIGO stage IIa, 20 FIGO stage IIIb, 8 FIGO stage IVb. Among them, 15 cases were pathologically characterized as well-differentiated, 13 moderately differentiated and 22 poorly differentiated tumors. Lymph node metastasis was documented for 26 tumor patients. The mean age of cervical cancer women was 52.7 with extreme ages at 39 and 67. Of patients with CIN enrolled for this study, including 20 cases with CIN II and 26 with CIN III. The median age of the CIN patients was 46.3 years, with a range of 29 to 56 years.

A total of 100 frozen biopsies tissue specimens consisting of 50 cases CSCC and 50 cases matched normal mucous epithelia (5 cm away from the tumor) were collected within 30 min after resection and kept at −80°C until used to determine tissue riboflavin levels and to determine C20orf54 mRNA and protein levels in 22 pairs of fresh CSCC.

A total of 146 of Peripheral blood samples were also collected from patients with CSCC, CIN, and controls, which used to measure blood riboflavin levels. The blood samples collected into EDTA Vacutainer Tubes and immediately placed on ice. Next, the remaining samples were centrifuged (10 min at 2000× g and 4°C) and collected plasma was stored at −80 °C until use. Control samples (n = 50) were obtained from subjects who underwent routine health examinations, were recruited in the same area, and were age-matched with CSCC patients. Selection criteria included individuals who were free from certain diseases including neoplasms, cardiovascular diseases, hepatic diseases, renal diseases, and inflammatory diseases.

### Determination of Riboflavin Levels in Plasma

Riboflavin in blood plasma was analyzed by high-performance liquid chromatography (HPLC). Use of HPLC as a method for separation and measurement of vitamins in plasma has been previously reported [25]–[27]. The HPLC system used was a Waters 2695 liquid chromatograph and Waters 2475 fluorescence detector and autosampler set at 28°C and configured for a 96-well microtiter plate. Water was purified using a Milli-Q water system. All chemicals were of analytical grade. For quality control, we used three Clin Chek serum controls, reconstituted and stored at −80°C. Aliquots of aqueous (0.3860 g/L C_2_H_7_NO_4_) flavin stock solutions (5 mmol/L) were stored at −20°C in the dark. Riboflavin was excited at 450 nm and detected at 520 nm, and the peak area was measured and used for quantification.

### Determination of Riboflavin Levels in Tissue

Tissue was analyzed for its concentration of riboflavin by an enzyme-linked immunosorbent assay (ELISA). Use of ELISA as a convenient method for measurement of riboflavin in tissue has been reported in a recent study by Wang *et al.* [28]. In accordance with ELISA kit instructions, it was used to detect the riboflavin levels in fresh tissue from cervical carcinoma patients and cervical mucosa from control tissue. Fresh tissue (100 mg) from the same patient with cervical cancer and cervical mucosa 5 cm away from the tumor (control group) were weighed on an electronic balance, added to l ml of 10 mm PBS solution, then homogenized. The homogenate was centrifuged 20 min at 2000 rpm, and 200 µl of supernatant was removed for analysis. The optical density (OD) of the supernatant was read within 15 min at 450 nm. A standard curve was used to determine the concentration of the sample.

### RNA Isolation and Real-time Quantitative PCR (qRT-PCR)

Total RNA was extracted from fresh frozen tissue using Trizol (Invitrogen, Carlsbad, CA, USA) according to the manufacturer’s instructions and treated with TURBODNA-freeTM DNase (Ambion, Austin, TX, USA) to remove the genomic DNA. Reverse transcription was performed using a reverse transcription system kit (Takara Bio, Tokyo, Japan). Real-time PCR for C20orf54 was performed in a 10-ul reaction volume using the Platinum SYBR Green qPCR SuperMix-UDG (Invitrogen Life Technologies, Carlsbad, CA, USA) and the Light Cycler 480 system (Roche Diagnostics, Penzberg, Germany). The following C20orf54 and β-actin (as a reference) primers were used for RT-PCR: C20orf54 forward primer: AATCTAGAGCACTTGGACCTTTCC; C20orf54 reverse primer GGG TTCAGGGACAGGTCTAAAGA; β-actin forward primer: GGCACCCAGCACAA TGAAG; β-actin reverse primer: CCGATCCACACGGAGTACTTG. The thermal cycle conditions were 95°C for 10 s for one cycle, followed by 40 cycles of amplification at 95°C for 5 s, and 60°C for 45 s. The expression level of C20orf54 mRNA was obtained using the 2-DDCT calculation method. All PCR products were analyzed on a 2% agarose gel with ethidium bromide staining.

### Protein Extraction and Western Blotting Analysis

Total protein from the same patient with fresh tissue of cervical cancer and cervical mucosa 5 cm away from the tumor tissue extracted with radio immunoprecipitation assay (RIPA) lysis buffer (Bioteke, Beijing, china) containing protease inhibitor. The proteins were separated by 10% SDS-PAGE (Invitrogen, Carlsbad, CA, USA) and transferred onto polyvinylidene difluoride (PVDF) membranes (Millipore, Billerica, MA). The membranes were incubated in blocking buffer (1 h with 5% skimed milk in PBST) at room temperature with gently shaking. Next the sample was incubated overnight at 4°C with primary antibody for anti-C20orf54 (Santa Cruz Biotechnology, Santa Cruz, CA, USA). After washing with PBST thrice, the membranes was incubated with horseradish peroxidase conjugated IgG at room temperature for 2 h. The blot was visualized using DAB kit (Zhongshan jinqiao, Beijing, China). Western blotting band was quantified using Quantity One software by measuring the band intensity for each group and normalizing to β-tubulin (Sigma) as internal control (Invitrogen). The final results were expressed as fold changes by normalizing date to the control values.

### Immunohistochemical Determination of C20orf54

Immunohistochemistry (IHC) was performed using Histostain-SP kits (Zhongshan Golden Bridge, Beijing, China) according to the manufacturer’s instructions that have been previously described [16]. Sections of paraffin embedded tissue (3 µm) were deparaffinized in xylene, then rehydrated in graded concentrations of ethyl alcohol (100%, 95%, 80%, and 70%), and pretreated in a microwave for 15 min on high mode in Tris/EDTA buffer (pH 9.0). After cooling and rinsing in distilled water, endogenous peroxidase activity was blocked with 3% hydrogen peroxide for 15 min at room temperature. Subsequently, the slides were pretreated with 1% bovine serum albumin in phosphate buffered saline (PBS, pH 7.4) for 10 min. Samples were then preincubated with a protein blocking solution for 15 min and incubated with a C20orf54 antibody (Santa Cruz Biotechnology, Santa Cruz, CA, USA) at a 1∶300 dilution in PBS at 4°C overnight in a humid chamber. Slides were washed three times in PBS, then incubated with a secondary biotinylated antibody for 15 min at room temperature. Thereafter, sections were washed with PBS, and then treated with peroxidase-conjugated streptavidin for 15 min. Finally, the sections were lightly stained with hematoxylin. PBS was used instead of the primary antibody as a negative control. All immunostained sections were coded and independently examined by two investigators using light microscopy. Results were scored on a scale from 0 to 3 by the percentage and intensity of positive cells among tumor cells as previously described [29].

The percentage of positive cells was scored as follows: 0 for ≤25%, 1 for 26–50%, 2 for 51–75%, and 3 for ≥76%. The intensity of staining was as follows: 0 indicated an absence of staining, 1 indicated weak staining, 2 indicated moderate staining, and 3 indicated intense staining. The sum of both scores was used to classify four categories of expression: strong expression (5–6), medium expression (3–4), weak expression (1–2), and total loss of expression (0).

### HPV Detection and Typing

For DNA extraction from formalin-fixed, paraffin-embedded CIN and CSCC tissues, each sample (2, 5 µm sections) were treated with 0.8 ml of lemosol and 0.2 ml of ethanol, and washed with 1 ml of ethanol. After centrifugation and air drying, the pellet was resuspended in digestion buffer (50 mM Tris-HCl, 1 mM EDTA, and 0.5% Tween 20, pH 8.0) containing 200 mg/ml of proteinase K (Invitrogen Corp, Carlsbad, CA, USA) and incubated for 24 h at 56°C. The solution was heated at 100°C for 10 min, followed by phenol–chloroform extraction and ethanol precipitation of DNA [30]. HPV genotyping was performed using reverse hybridization using the Human Papillomavirus Genotyping Diagnosis Kit (Genetel Pharmaceuticals, Shenzhen, China) and analyzed using the HPV genotype DNA microarray reader system (HPV-GenoCam-9600, Genetel Pharmaceuticals). The diagnostic test contained probes for 29 HPV genotypes (HPV16, 18, 31, 33, 35, 39, 45, 51, 52, 56, 58, 59, 68, 6, 11, 26, 40, 42, 43, 44, 53, 54, 55, 57, 66, 67, 69, 73, and 82). The hybridization steps were performed following the manufacturer’s instructions [31]. Briefly, HPV DNA was extracted and PCR amplified using the following parameters: denaturation at 9°C for 10 min, 40 cycles of 95°C for 30 seconds, 52°C for 45 sec, and 65°C for 90 sec. At the end of the last cycle, the mixture was incubated at 65°C for 5 min. The gene chip was prepared, and the PCR products were hybridized and visualized.

### Statistical Analysis

All statistical analyses were performed with SPSS Version 17 software. *P* values were two-sided, and the significance level was *P*<0.05. The Mann-Whitney test was used to test continuous variables for differences in immunohistochemical staining scores between tumor and normal tissues for C20orf54. Differences of riboflavin levels in plasma and tissue between subgroups were tested with the Student’s *t*-test. Results were presented as mean ± SD. Variance analysis were used to analyze the association between two continuous variables.

## Results

### Analyses of Riboflavin Levels in Plasma of CSCC Patients, CIN Patients, and Controls

**Figure 1:**
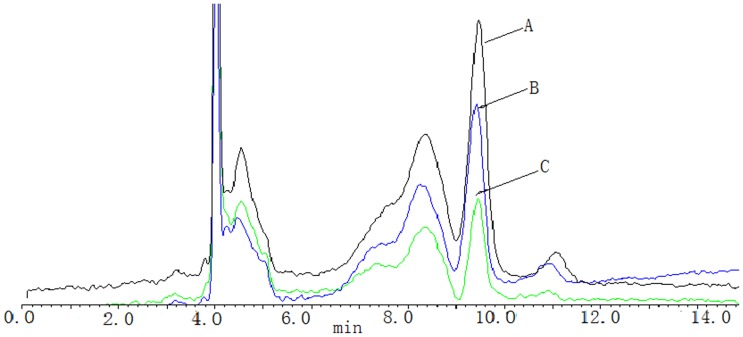
Chromatographic profiles of riboflavin in a CSCC patient, a CIN patient and a normal control, respectively. Retention times for riboflavin were approximately 9.75 min. A for a normal control, B for CIN, C for a CSCC.

**Figure 2:**
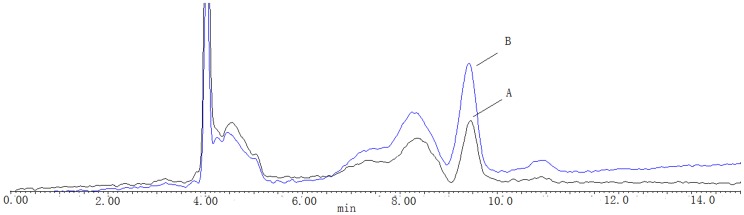
Chromatographic profiles of riboflavin in a low tumor stage patient and a high tumor stage patient.

Riboflavin concentrations were determined in samples obtained from cervical cancer patients who were not taking vitamin supplements. Sirocco- ^TM^96 precipitation plate and the positive pressure -96 processor were used for sample pretreatment. The chromatographic column was symmertryshield™ RP-C_18_ (250 mm×4.6 mm, 5 um), the mobile phase was 35% methanol and 65% 5 mmol·L^−1^ ammonium acetate solution at a flow rate of 0.6 ml·min^−1^.The spectro-photofluorimeter was set at wave-length of 450 nm for excitation and 520 nm for emission. The linear ranges were 0.2–10 ng· mL^−1^(r = 0. 9937). The lowest limit of quantification was 0. 17 ng·mL^−1^. The intra - day RSD were 2. 0%–4.8%, the intra - day RSD was 3.8%. The relative recoveries were between 101.5% and 116. 0%(n = 3).The recovery of extraction was 91.8%–115.1%.Riboflavin chromatograms of plasma samples from a CIN patient and a CSCC patient, and a controls are shown in Fig. 1. The plasma riboflavin levels in patients with CSCC were significantly lower than those in the healthy controls and CIN patients (254.62±98.36 µg/L vs. 310.01±90.15 µg/L; 254.62±98.36 µg/L *vs*. 294.62±88.36 µg/L, respectively, all *P*<0.05). The average blood riboflavin level in high tumor stage patients (≤IIa) was 182.01±89.43 ug/L, which was significantly lower than in patients with low tumor stage ≥IIb (277.25±34.56 µg/L). Riboflavin chromatograms of plasma samples from a low tumor stage patient and a high tumor stage patient are shown in Fig. 2.

### Analyses of Riboflavin Levels in Tissues of CSCC Patients and Their Matched Controls

The average riboflavin level in CSCC tissues was 15.12±3.72 µg/L, and the average level in tissues of matched normal cervical epithelial tissue was 20.12±4.61 µg/L. A decreased tissue riboflavin level was found to in CSCC compare with normal cervical epithelial tissue (Table 1).

**Table 1:** Comparison of riboflavin levels in plasma from CSCC patients, CIN patients, and control subjects, and in tissues of CSCC and normal cervical epithelia.

groups	riboflavin level in plasma (means ± SD ug/L)	*P*	riboflavin level in tissues (means ± SD ug/L)	*P*
Normal	310.01±90.15	0.034	20.12±4.61	0.002
CIN	294.62±88.36			
CSCC	254.62±98.36		15.12±3.72	
≤IIa	277.25±34.56	0.012	16.45±3.01	0.039
≥IIb	182.01±89.43		14.23±2.92	

Student’s *t*-test was used to Comparison of riboflavin levels in plasma from CSCC patients, CIN patients, and control subjects, and in tissues of CSCC and normal cervical epithelia.

### Analyses of C20orf54 at mRNA and Protein Expression Level

**Figure 3:**
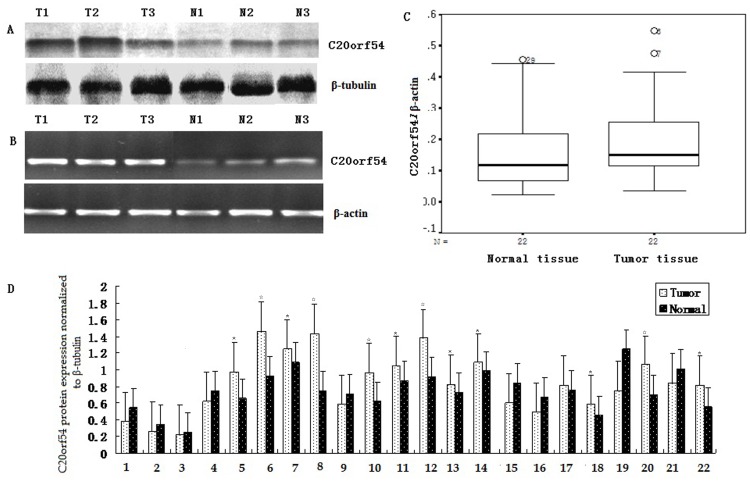
higher-expression of C20orf54 was detected in CSCC tissue by Western blot (A) and qRT-PCR (B). A, Representative results of C20orf54 and β-tubulin protein was described in CSCC (T) and matched normal tissues (N) by western blot. B, Representative results of C20orf54 and β-actin mRNA was examined in CSCC (T) and corresponding normal tissues (N) by qRT-PCR. C, Box plot, the expression of C20orf54 mRNA was significantly higher in CSCC than that matched tissue(0.21±0.14 *vs* 0.16±0.12; n = 22, *P*<0.05). D,Bar chart for relative expression of C20orf54 protein in CSCC and matched tissue(0.93±0.41 *vs* 16.13±3.88; n = 22, repeat three times in each sample,* indicates *P*<0.05, ☆indicates *P*<0.01).

After normalization with β-actin control in CSCC, Higher mRNA transcript of C20orf54 were displayed in CSCC with 72.7%(16/22) compared with control(*P*<0.05). In additional, based on western blot analysis, expression of C20orf54 protein were found to have a higher with 63.6%(14/22) in CSCC compare to matched tissues with 36.4%(8/22). The detailed results of C20orf54 mRNA and protein were summarized in Table 2. Statistical analysis demonstrated that expression of C20orf54 in CSCC was significantly higher than that in normal tissues at both mRNA and protein level (Fig. 3).

**Table 2:** The expression of C20orf54 in fresh CSCC and control on both mRNA and protein level.

	mRNA level	*P*	protein level	*P*
CSCC	0.21±0.14	0.013	0.93±0.41	0.045
Normal	0.16±0.12		0.80±0.36	
Number	22		22	

Student’s *t*-test was used to Comparison of C20orf54 mRNA and protein levels in tissue from CSCC patients and normal cervical epithelia.

### Correlation between Clinic-pathological Characteristics and C20orf54 Over Expression

**Figure 4:**
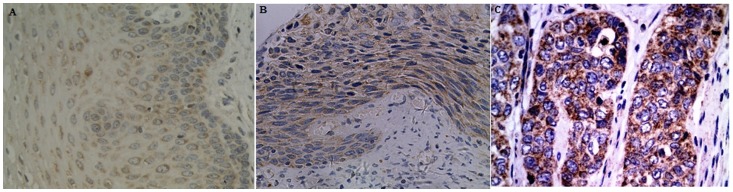
Immunohistochemical analyses. A–C Staining patterns of C20orf54 in normal cervical epithelia, CIN and CSCC, respectively. A: Expression of C20orf54 in normal cervical epithelia with weak staining; B: Moderate expression of C20orf54 protein in CIN tissue; C: Strong expression of C20orf54 protein in CSCC tissue (original magnification,×400).

IHC staining of primary CSCC lesions and samples adjacent to the tumor, and 46 CIN was performed using C20orf54 antibodies (Table 3). Representative staining patterns for C20orf54 are shown in Fig. 4. The staining showed that C20orf54 was localized to the cytoplasm. Positive staining for C20orf54 was generally observed within CSCC cells, but weak or no C20orf54 staining was detected in normal cervical epithelium adjacent to the tumor. C20orf54 expression was undetectable (36%) in normal cervical epithelia. In contrast, strong IHC staining (3+) was detected in cervical cancer tissue. The positive rates (strong staining, medium staining, and weak staining) of C20orf54 expression in cervical cancer were 48.0%, 28.0%, and 16.0%, respectively. We also evaluated the possible relationship between expression of C20orf54 in tumor cells and the clinicopathologic characteristics of CSCC, including tumor stage, histological grade, and lymph node metastasis. C20orf54 expression was significantly increased in CSCC with high tumor stage. However, other parameters such as lymph node metastasis and histological grade had no significant relationship with C20orf54 expression (Table 3).

**Table 3:** Statistical analysis of C20orf54 expression and clinicopathologic factors in CIN and cervical cancer.

Characteristics	N	C20orf54 expression				Z	*P*
		−	+	++	+++		
Normal mucousepithelia	50	18	25	7	0	42.485	<0.001*
CIN II–III	46	14	15	12	5		
CSCC	50	4	8	14	24		
Differentiation							
Moderate/well	22	2	3	7	10	−1.238	0.216
Poor	28	2	5	7	14		
L/N metastasis							
Negative	24	1	6	3	14	−0.164	0.87
Positive	26	3	2	11	10		
Stage							
II and IIIa	21	3	6	8	4	−2.625	0.009
IIIb and IV	29	1	2	6	20		

L/N metastasis: Lymph node metastasis. −: Negative;+: Weak positive;++: Medium positive;+++: Strong positive. Mann-Whitney test were used to test continuous variables for differences in IHC staining scores between tumor and normal tissues for the C20orf54. * was shown for comparison with CIN, CSCC and normal control groups.

### Type Prevalence of HPV

Of the 96 samples processed for HPV testing, no HPV DNA was detected in 27 specimens, 9 of which were from invasive cancers. From the 69 HPV positive specimens, 13 HPV genotypes were detected. HR-HPV genotypes included HPVl6,18,31,33,35,39,45,51,52,56,58,59,67,68,73, and 82. LR-HPV genotypes include HPV44 and 55. HR-HPV was detected in 86.6% of CIN specimens and 85.7% of specimens with invasive cancer, and LR-HPV was detected in 7.0% of CIN specimens and 3.5% of specimens with invasive cancer. The specific prevalence of HPV 16, 18, 51, 55, 31, 44, and 33 were 63.2%, 10.5%, 8.8%, 7.0%, 5.3%, 3.5%, and 1.8%, respectively. HPV type 16 was the most common in all diagnosis categories. Co-infection with HR-HPV and HR/LR-HPV were 15.9%, and 1.4%, respectively. HPV 16/82 and HPV 16/18 co-infections were detected in 25.0% of the co-infection group, respectively.

### Association of the Riboflavin Levels with C20orf54 Protein Expression and HPV16/18 and with 16/18 Co-infection

We analyzed the relationship between tissue riboflavin levels and expression of the C20orf54 gene and development of CSCC. A negative association was found between changes in tissue riboflavin levels and changes in C20orf54 protein expression (Table 4). We further analyzed the relationship between tissue riboflavin levels and HPV16, HPV 18 (and with 16/18 co-infection) infection in CSCC, due to the HPV type 16 and 18 were the most common in all diagnosis categories. The results showed that tissue riboflavin level was significantly lower in HPV16, HPV 18, and with 16/18 co-infection positive tissue than negative tissue (16.79±3.01 µg/L *vs*. 14.03±3.76 µg/L).

**Table 4:** Statistical analysis of C20orf54 protein expression and tissue riboflavin levels in CSCC and matched normal tissues.

	CSCC(n = 22,mean ± SD)	t	*P*
C20orf54 protein expression	0.93±0.41	−2.125	0.029
Riboflavin level in tissue	16.13±3.88		

Student’s *t*-test was used to Comparison of C20orf54 protein levels in tissue with tissue riboflavin levels from CSCC patients.

## Discussion

This study demonstrated that samples of recurrent Uyghur CSCC and CIN exhibited significantly higher protein levels of C20orf54 than normal counterpart tissue. There was also an inverse association between tissue and plasma riboflavin levels and C20orf54 expression with CSCC risk. A positive association was found between tissue and plasma riboflavin levels and C20orf54 with CSCC risk. There was also an inverse association between riboflavin levels and HPV16, HPV 18, and (with 16/18 co-infection) infection. The results indicated that C20orf54 likely plays an important role in Uyghur cervical carcinogenesis by modulating riboflavin absorption and by affecting the persistence of HPV infection that influences progression of early precancerous lesions to invasive cancer.

Previous studies have reported that riboflavin deficiency can cause a variety of metabolic problems that can lead to skin and mucosal disorders. Riboflavin is a precursor for flavin adenine dinucleotide, a cofactor for the critical folate dependent enzyme methylenetetrahydrofolate reductase (MTHFR). Furthermore, folate plays a key role in DNA synthesis and methylation, and it is essential in maintenance of DNA integrity, stability, and repair [32]. Thus, riboflavin intake influences functional folate status [33]. Studies have also examined the possible contribution that riboflavin might make to protection against cancer. Piyathilake CJ *et al.* [34] reported that riboflavin modified MTHFR polymorphism in cervical intraepithelial neoplasia. The present study reported riboflavin concentrations determined by HPLC in plasma samples from CIN and CSCC patients, as well as riboflavin determined by ELISA in tissue samples from CSCC patients and matched normal mucous epithelial tissue. The results showed that riboflavin levels not only decreased in plasma from CIN and CSCC patients compared with control subjects, but also decreased in CSCC tissue compared with normal tissue. Although tumor tissue is restricted to certain organs, cancer is believed to be a disease of the host when the whole body is switched to a pathological state. As a result, plasma from cancer patients contains all the information about pathogenic changes in certain organs or tissues. Therefore, the results suggested that riboflavin likely plays an important role in cervical carcinogenesis. Previous studies have also reported that riboflavin is a potential cancer preventive agent because of its role in one carbon metabolism [35]. Singh *et al.* [36] reported that a reduced riboflavin intake can lead to changes in the methyl supply, followed by alterations in DNA methylation. Recent epidemiological studies of 718 Chinese breast cancer cases showed that low intakes of riboflavin are associated with breast carcinogenesis [37]. There are also studies of cancer cell lines showing that riboflavin can activate extrinsic apoptosis pathways at low concentration. Additional cell death mechanisms like intrinsic apoptotic pathways and protease pathways are also triggered at higher concentrations of riboflavin, leading to further inhibition in their proliferation [14], [38].

It is well established that milk and dairy products, meats, fatty fish, and certain fruits and vegetables are good sources of riboflavin. Humans are unable to synthesize riboflavin and thus must acquire it from exogenous sources in the diet. Therefore, it is important whether the host can effectively absorb and utilize riboflavin from foods. However, riboflavin deficiency and poor riboflavin status seem to be frequent in cancer patients, despite the diversity of available riboflavin-rich foods. Thus, whether other factors exist that may influence dietary riboflavin absorption or that may change the status of riboflavin needs to be determined. To clarify these issues, we characterized the riboflavin transporter 2 (C20orf54) that modulates riboflavin absorption. The results showed that C20orf54 protein expression gradually increased in CIN and CSCC. Furthermore, it is inconsistent that in gastric cancer, the riboflavin transporter 2 protein shows reduced expression [39]. In normal conditions, C20orf54 was not only expressed in small intestine but also expressed in jejunum, ileum, and testis, as well as in lung, kidney, stomach, and colon, but scarcely expressed in other organs or tissues [40].

Based on our results, we conclude that C20orf54 not only plays an important role in intestinal riboflavin absorption, but also affects riboflavin tissue distribution of riboflavin, because after absorption, riboflavin is distributed from the blood to several tissues, and utilized as a coenzyme in metabolic reactions. This is consistent with the present study, which shows an inverse association between tissue riboflavin levels and C20orf54 protein expression in CSCC. Therefore, C20orf54 expressed in cervical tissue could play a key role in riboflavin transport.

Persistent infection with oncogenic high risk HPV types, especially 16 and/or 18, accounts for the majority of cervical cancer and is associated with intraepithelial neoplasia. However, HPV infection is usually transient, and only a small proportion of women who test positive for high risk HPV infection develop cervical cancer. Therefore, nutritional factors may affect the persistence of HPV infection and thereby influence progression of early precancerous lesions to invasive cancer. In the present study, a high frequency of HPV-16 infection, followed by HPV-18 infection, was observed in our survey. Therefore, we analyzed the association of tissue riboflavin levels and HPV16, HPV 18 (and with 16/18 co-infection) infection in CSCC. The results showed that plasma and tissue riboflavin levels were inversely associated with HPV16 and HPV 18 infection. It has been reported that micronutrients and vitamin might limit HPV infection through antioxidant activity, because antioxidant activity was shown to reduce HPV transcription in the cervical cell line HeLa, and HPV-16 expression was also shown to be modulated by redox status in vitro [41]–[42], and similar studies have also been reported for plasma folate after HPV16 infection [43]–[47].

Our study has some limitations. It includes possible selection bias, recall bias with respect to reporting of dietary intake, and the possibility that patients might have changed their diet with the onset of disease. In addition, plasma riboflavin levels are transient and depend on recent, rather than long-term dietary riboflavin intake, so there may be poor correlations of present plasma riboflavin levels to lifetime exposure. However, the present study also has several advantages including the first report of associations of riboflavin levels in tissue and plasma with riboflavin transporter gene (C20orf54) expression in CSCC. In addition, although plasma riboflavin levels are transient and dependent on recent, rather than long-term dietary riboflavin intake, we further analyzed tissue riboflavin levels that reflected C20orf54 function. These results are also supported by the observation that riboflavin transporter 2 (C20orf54) was highly expressed in the small intestine and involved uptake of riboflavin in the small intestine for nutritional utilization [16].

Evidence for a protective effect of riboflavin against cervical cancer is currently weak, but this may be partly due to a failure to consider the role of HPV infection as a key risk factor. Riboflavin may modulate HPV persistence and thereby influence cancer risk. In the present study, we provided little evidence for insufficient riboflavin was associated with an increased risk of cervical dysplasia and persistence of HPV infection. Furthermore, C20orf54 may contribute to modulating riboflavin absorption in Uyghur cervical carcinogenesis.

However, given the potential role of these nutrients in carcinogenesis and particularly given the paucity of studies to date regarding intake of riboflavin and the risk of cervical epithelial cancers, further investigations are warranted.

## References

[1] International Agency on Research on Cancer (IARC) (1995) Monograph on cancer, Human papillomavirus. Vol : 64. World Health Organization.

[2] Powers HJ (2005) Interaction among folate, riboflavin, genotype, and cancer with reference to colorectal and cervical cancer. J Nutr 135: 2960S–2966S. 16317155 10.1093/jn/135.12.2960S

[3] Castellsague X, Munõz N (2003) Cofactors in human papillomavirus carcinogenesis- role of parity, oral contraceptives, and tobacco smoking. J Natl Cancer Inst Monogr 31: 20–28. 12807941

[4] Kabat GC, Miller AB, Jain M, Rohan TE (2008) Dietary intake of selected B vitamins in relation to risk of major cancers in women. British Journal of Cancer 99: 816–821. 18665162 10.1038/sj.bjc.6604540 PMC2528139

[5] Potischman N, Brinton LA (1996) Nutrition and cervical neoplasia. Cancer Causes Control 7: 113–126. 8850440 10.1007/BF00115643

[6] Steinmetz K, Potter JD (1996) Vegetables, fruit and cancer prevention: a review. J Am Diet Assoc 96: 1027–1037. 8841165 10.1016/S0002-8223(96)00273-8

[7] Ziegler RG, Lim U (2007) One-carbon metabolism, colorectal carcinogenesis, chemopreventio n–with caution. J Natl Cancer Inst 99: 1214–1215. 17686821 10.1093/jnci/djm105

[8] Mason JB (2003) Biomarkers of nutrient exposure and status in one-carbon (methyl) metabolism. J Nutr l 3: S941–947. 10.1093/jn/133.3.941S 12612180

[9] De Souza ACS, Kodach L, Gadelha FR, Bos CL, Cavagis ADM, et al (2006) A promising action of riboflavin as a mediator of leukaemia cell death. Apoptosis 11: 1761–1771. 16927017 10.1007/s10495-006-9549-2

[10] Kim YI (2004) Folate and DNA methylation: a mechanistic link between folate deficiency and colorectal cancer? Cancer Epidemiol Biomarkers Prev 13: 511–519. 15066913

[11] Webster RP, Gawde MD, Bhattacharya RK (1996) Modulation of carcinogen induced DNA damage and repair enzyme activity by dietary riboflavin. Cancer Lett 98: 129–135. 8556699

[12] Zhu X, Wentworth P, Kyle RA, Lerner RA, Wilson IA (2006) Cofactorcontaining antibodies: Crystal structure of the original yellow antibody. Precede Natr Acad Sci 103: 3581–3585. 10.1073/pnas.0600251103 PMC1450126 16537445

[13] Santos NAG, Catao CS, Martins NM, Curti C, Bianchi MLP, et al (2007) Cisplatin induced nephrotoxicity is associated with oxidative stress, redox state unbalance, impairment of energetic metabolism and apoptosis in rat kidney mitochondria. Arch Toxicol 81: 495–504. 17216432 10.1007/s00204-006-0173-2

[14] De Souza QKC, Zambuzzi WF, De Souza ACS, Da Silva RA, Machado D, et al (2007) A possible anti-proliferative and anti-metastatic effect of irradiated riboflavin in solid tumors. Cancer Lett 258: 126–134. 17933458 10.1016/j.canlet.2007.08.024

[15] Yamamoto S, Inoue K, Ohta KY, Fukatsu R, Maeda JY, et al (2009) Identification and functional characterization of rat riboflavin transporter 2.J Biochem. 145: 437–443. 10.1093/jb/mvn181 19122205

[16] Misaki F, Syunsuke Y, Tomoaki M, Tomoya Y, Katsuhisa I, et al (2010) Functional Characteristics of the Human Ortholog of Riboflavin Transporter 2 and Riboflavin-Responsive Expression of Its Rat Ortholog in the Small Intestine Indicate Its Involvement in Riboflavin Absorption. J Nutr 140: 1722–1727. 20724488 10.3945/jn.110.128330

[17] Siassi, F Ghadirian (2005) P (2005) Riboflavin deficiency and esophageal cancer: a case control-household study in the Caspian Littoral of Iran. Cancer Detect. Prev 29: 464–469. 10.1016/j.cdp.2005.08.001 16183212

[18] He Y, Ye L, Shan B, Song G, Meng F, et al (2009) Effect of riboflavin-fortified salt nutrition intervention on esophageal squamous cell carcinoma in a high incidence area, China. Asian Pac J Cancer Prev10: 619–622. 19827881

[19] Yang B, Bray F, Parkin D, Sellors J, Zhang Z (2004) cervical cancer as a priority for prevention in different world regions: an evaluation using years of life lost. Int J Cancer 109: 418–424. 14961581 10.1002/ijc.11719 PMC4167424

[20] Lalai S, Yu-Hua P, Fu-sheng Zh (2006) Analysis of the cervical cancer distribution in Xinjiang. Journal of Xinjiang Medical University 29: 569–571 (Article in Chinese)..

[21] Brock KE, Berry G, Mock PA, MacLennan R, Truswell AS, et al (1988) Nutrients in diet and plasma and risk of in situ cervical cancer. JNatl Cancer Inst 80: 580–585. 3373548 10.1093/jnci/80.8.580

[22] Hernandez BY, McDuffie K, Wilkens LR, Kamemoto L, Goodman MT (2003) Diet and premalignant lesions of the cervix: evidence of a protective role of folate, riboflavin, thiamine and vitamin B12. Cancer Causes Control 14: 859–870. 14682443 10.1023/b:caco.0000003841.54413.98

[23] Murphy SJ, Anderson LA, Ferguson HR, Johnston BT (2010) Dietary antioxidant and mineral intake in humans is associated with reduced risk of esophageal adenocarcinoma but not reflux esophagitis or Barrett’s esophagus. J Nutr 140: 1757–1763. 20702746 10.3945/jn.110.124362

[24] Liu T, Soong SJ, Wilson NP, Craig CB, Cole P, et al (1993) A case control study of nutritional factors and cervical dysplasia. Cancer Epidemiol Biomarkers Prev 2: 525–230. 8268768

[25] Petteys BJ, Frank EL (2011) Rapid determination of vitamin B_2_ (riboflavin) in plasma by HPLC. Clin Chim Acta 412: 38–43. 20816949 10.1016/j.cca.2010.08.037

[26] Tuan PA, Kim JK, Lee S, Chae SC, Park SU (2012) Riboflavin Accumulation and Characterization of cDNAs Encoding Lumazine Synthase and Riboflavin Synthase in Bitter Melon. J Agric Food Chem 60: 11980–11986. 23153065 10.1021/jf3036963

[27] Shen Y, Zhang P, Kong X, Guo C, Wang J (2005) Simultaneous determination of water-soluble vitamins C, B1, B2 and B6 in almonds by high performance liquid chromatography. Se Pu 23: 538–541. 16350802

[28] ai-fang J, wu W, jin-sheng W, li-dong W (2012) Comparison of blood and tissue riboflavin levels between esophageal squamous cell carcinoma and healthy control. Cancer Research on Prevention and Treatment 39: 467–469 (Article in Chinese)..

[29] Ayshamgul H, Mangnishahan A, Jun-Qi M, Zhen J, Gulzareye A, et al (2012) Post-transcriptional and Epigenetic Regulation of Antigen Processing Machinery (APM) components and HLA-I in Cervical Cancers from Uighur Women. PLoS ONE 9: e44952. 10.1371/journal.pone.0044952 PMC3443204 23024775

[30] Khan NA, Castillo A, Koriyama C, Kijima Y, Umekita Y, et al (2008) Human papillomavirus detected in female breast carcinomas in Japan. British Journal of Cancer 99: 408–414. 18648364 10.1038/sj.bjc.6604502 PMC2527789

[31] Corneanu LM, Stănculescu D, Corneanu C (2011) HPV and cervical squamous intraepithelial lesions: clinicopathological study. Rom J Morphol Embryol 52: 89–94. 21424037

[32] Choi SW, Mason JB (2000) Folate and carcinogenesis: an integrated scheme. J Nutr130: 129–132. 10.1093/jn/130.2.129 10720158

[33] Van den Donk M, Buijsse B, van den Berg SW, Ocke MC, Harryvan JL, et al (2005) Dietary intake of folate and riboflavin, MTHFR C677T genotype, and colorectal adenoma risk: a Dutch case-control study. Cancer Epidemiol Biomarkers Prev 14: 1562–1566. 15941973 10.1158/1055-9965.EPI-04-0419

[34] Piyathilake CJ, Azrad M, Macaluso M, Johanning GL, Cornwell PE (2007) Protective association of MTHFR polymorphism on cervical intraepithelial neoplasia is modified by riboflavin status. Nutrition 23: 229–235. 17303386 10.1016/j.nut.2006.12.006 PMC2025704

[35] Selhub J (2002) Folate, vitamin B12 and vitamin B6 and one carbon metabolism. J Nutr Health Aging 6: 39–42. 11813080

[36] Singh SM, Murphy B, O’Reilly RL (2003) Involvement of gene-diet/drug interaction in DNA methylation and its contribution to complex diseases: from cancer to schizophrenia. Clin Genet 64: 451–460. 14986824 10.1046/j.1399-0004.2003.00190.x

[37] Shrubsole MJ, Shu XO, Li HL, Cai H, Yang G, Gao YT, et al (2011) Dietary B vitamin and methionine intakes and breast cancer risk among Chinese women. Am J Epidemiol 173: 1171–1182. 21447479 10.1093/aje/kwq491 PMC3121320

[38] Hassan I, Chibber S, Khan AA, Naseem I (2012) Riboflavin Ameliorates Cisplatin Induced Toxicities under Photoillumination. PLoS ONE 7: e36273. 22567145 10.1371/journal.pone.0036273 PMC3342168

[39] Maynur E, De-sheng L, Wei-wei Zh, Bing K, Ilyar Sh, et al (2012) Decreased blood riboflavin levels are correlated with defective expression of the riboflavin transporter 2 (RFT2) gene in GC. World Journal of Gastroenterology 18: 3112–3118. 22791947 10.3748/wjg.v18.i24.3112 PMC3386325

[40] Yamamoto S, Inoue K, Ohta KY, Fukatsu S, Maeda JY, et al (2009) Identification and Functional Characterization of Rat Riboflavin Transporter 2. J Biochem 145: 437–443. 19122205 10.1093/jb/mvn181

[41] Prusty BK, Das BC (2005) Constitutive activation of transcription factor AP-1 in cervical cancer and suppression of human papillomavirus (HPV) transcription and AP-1 activity in HeLa cells by curcumin. Int J Cancer 113: 951–960. 15514944 10.1002/ijc.20668

[42] Rosl F, Das BC, Lengert M, Geletneky K, zur Hausen H (1997) Antioxidantinduced changes of the AP-1 transcription complex are paralleled by a selective suppression of human papillomavirus transcription. J Virol 71: 362–370. 8985358 10.1128/jvi.71.1.362-370.1997 PMC191059

[43] Piyathilake CJ, Macaluso M, Brill I, Heimburger DC, Partridge EE (2007) Lower red blood cell folate enhances the HPV-16-associated risk of cervical intraepithelial neoplasia. Nutrition 23: 203–210. 17276035 10.1016/j.nut.2006.12.002

[44] Sedjo RL, Inserra P, Abrahamsen M, Harris RB, Roe DJ, et al (2002) Human papillomavirus persistence and nutrients involved in the methylation pathway among a cohort of young women. Cancer Epidemiol Biomarkers 11: 353–359. 11927495

[45] Hernandez BY, McDuffie K, Wilkens LR, Kamemoto L, Goodman MT (2003) Diet and premalignant lesions of the cervix: evidence of a protective role for folate, riboflavin, thiamin, and vitamin B12. Cancer Causes Control 14: 859–870. 14682443 10.1023/b:caco.0000003841.54413.98

[46] Yonezawa A, Masuda S, Katsura T, Inui K (2008) Identification and functional characterization of a novel human and rat riboflavin transporter, RFT1. Am J Physiol Cell Physiol 295: C632–C641. 18632736 10.1152/ajpcell.00019.2008

[47] Fujimura M, Yamamoto S, Murata T, Yasujima T, Inoue K, et al (2010) Functional characteristics of the human ortholog of riboflavin transporter 2 and riboflavin-responsive expression of its rat ortholog in the small intestine indicate its involvement in riboflavin absorption. J Nutr140: 1722–1727. 10.3945/jn.110.128330 20724488

