# Correction: Association of the Plasma and Tissue Riboflavin Levels with C20orf54 Expression in Cervical Lesions and Its Relationship to HPV16 Infection

**DOI:** 10.1371/journal.pone.0103377

**Published:** 2014-07-16

**Authors:** The PLOS ONE Staff

In the first paragraph of the Results section, there is an error in the penultimate sentence. Please see the corrected sentence below:

“The average blood riboflavin level in high tumor stage patients ( ≥IIb) was 182.01±89.43 ug/L, which was significantly lower than in patients with low tumor stage ≤IIa(277.25±34.56 ug/L)."

There is an error in the Figure 3 legend. Please see the figure and the corrected legend below:

**Figure 3:**
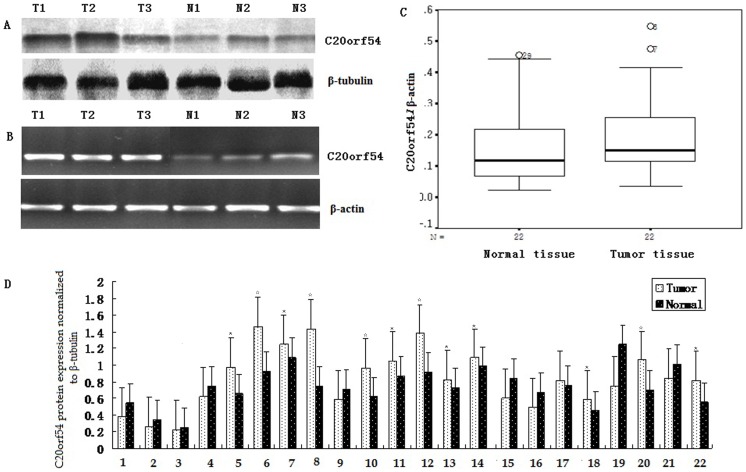
higher-expression of C20orf54 was detected in CSCC tissue by Western blot (A) and qRT-PCR (B). A, Representative results of C20orf54 and β-tubulin protein was described in CSCC (T) and matched normal tissues (N) by western blot. B, Representative results of C20orf54 and β-actin mRNA was examined in CSCC (T) and corresponding normal tissues (N) by qRT-PCR. C, Box plot, the expression of C20orf54 mRNA was significantly higher in CSCC than that matched tissue(0.21±0.14 vs 0.16±0.12; n  =  22, P<0.05). D, Bar chart for relative expression of C20orf54 protein in CSCC and matched tissue (0.93± 0.41 vs 0.80±0.36; n  =  22) repeat three times in each sample,* indicates P<0.05, ☆indicates P<0.01).
